# Healthcare utilisation in the last year of life in internal medicine, young-old versus old-old

**DOI:** 10.1186/s12877-020-01894-0

**Published:** 2020-11-23

**Authors:** Vanda Ho, Cynthia Chen, Sara Ho, Benjamin Hooi, Loo Swee Chin, Reshma Aziz Merchant

**Affiliations:** 1 Division of Advanced Internal Medicine, Department of Medicine, National University Hospital, Singapore, Singapore; 2 Saw Swee Hock School of Public Health, National University Singapore, Singapore, Singapore; 3 Department of Medicine, Yong Loo Lin School of Medicine, National University Singapore, Singapore, Singapore

**Keywords:** End-of-life, Healthcare utilisation, Older adults

## Abstract

**Background:**

With increasing cost of healthcare in our aging society, a consistent pain point is that of end-of-life care. It is particularly difficult to prognosticate in non-cancer patients, leading to more healthcare utilisation without improving quality of life. Additionally, older adults do not age homogenously. Hence, we seek to characterise healthcare utilisation in young-old and old-old at the end-of-life.

**Methods:**

We conducted a single-site retrospective review of decedents under department of Advanced Internal Medicine (AIM) over a year. Young-old is defined as 65–79 years; old-old as 80 years and above. Data collected was demographic characteristics; clinical data including Charlson Comorbidity Index (CCI), FRAIL-NH and advance care planning (ACP); healthcare utilisation including days spent in hospital, hospital admissions, length of stay of terminal admission and clinic visits; and quality of end-of-life care including investigations and symptomatic control. Documentation was individually reviewed for quality of communication.

**Results:**

One hundred eighty-nine older adult decedents. Old-old decedents were mostly females (63% vs. 42%, *p* = 0.004), higher CCI scores (7.7 vs 6.6, *p* = 0.007), similarly frail with lower polypharmacy (62.9% vs 71.9%, *p* = 0.01). ACP uptake was low in both, old-old 15.9% vs. young-old 17.5%. Poor prognosis was conveyed to family, though conversation did not result in moderating extent of care.

Old-old had less healthcare utilisation. Adjusting for sex, multimorbidity and frailty, old-old decedents had 7.3 ± 3.5 less hospital days in their final year. Further adjusting for cognition and residence, old-old had 0.5 ± 0.3 less hospital admissions. When accounted for home care services, old-old spent 2.7 ± 0.8 less hospital days in their last admission.

**Conclusion:**

There was high healthcare utilisation in older adults, but especially young-old. Enhanced education and goal-setting are needed in the acute care setting. ACP needs to be reinforced in acute care with further research to evaluate if it reduces unnecessary utilisation at end-of-life.

## Background

Globally, societal ageing is one of the most pressing concerns of our time [1]. Singapore’s life expectancy has increased dramatically from 67 years in 1965 to 83.2 years in 2018 [2]. She is one of the fastest aging populations in Asia, and is expected to take only 27 years to transit from an ‘ageing society’ (7% seniors in 1999) to a ‘super-aged society’ (20% seniors in 2026) [3]. In contrast, Japan, China, Germany and the United States are expected to take 32 to 86 years [4]. The longer life expectancy of Singaporeans comes with social and fiscal implications. The government health expenditure alone has increased by 335%, from $2 billion in 2006 to $8.7 billion in 2015 [5]. This is accompanied by the rising prevalence of disability, frailty, dementia and chronic diseases [6].

In addition, healthcare utilisation tends to increase towards the end-of-life [7–10] and most deaths still occur in hospital [11]. While our life expectancy is rising [12], our health span is not: 2017 data found male Singaporeans more likely to spend the last 8 years of life in ill health and females, the last 10 years [13]. There is a wide variation of disabilities in the period before death, as per sociologists Glaser and Strauss’ observation on dying trajectories and modelling of latent trajectory of disability [14]. This course of disability in the final years of life does not follow a predictable pattern unlike those with cancer [15], and so characterising the vulnerable population at risk of high care needs is paramount in targeting services appropriately.

In most countries, patients with multimorbidity and chronic diseases with or without functional needs are admitted under Internal Medicine. There are many studies focussing on end of life care in cancer patients but very few on those with non-cancer related illness and frailty [16]. It is often difficult to predict recovery in this group of patients [17] leading to possible increased healthcare utilisation without necessarily improving quality of care [18].

Due to the unpredictable trajectories for patients admitted under Internal Medicine, we aimed to study healthcare utilisation in the young-old and old-old non-cancer patients in the final year before death.

## Methods

We retrospectively examined case records from all patients 65 years old and above who died under the division of Advanced Internal Medicine in National University Hospital (NUH), Singapore from Jan to Dec 2015. Patients who were admitted directly into intensive care unit (ICU) from the emergency department (ED) and those who had an active cancer diagnosis were excluded. Patients with past cancer diagnosis that is considered cured or in remission remained in the study. During the year of data collection, there were 195 deaths. Six records were of patients younger than 65 years old and were excluded. As our study was looking at healthcare utilisation of complex and vulnerable older adult, with potential fiscal implications, we took the same cut-off as the old-age support ratio of Singapore of 65 years and above as older adult [19] to focus our analysis on the older adult population in our country. As per the United Nation’s agreed cut-off of oldest-old [20], we defined old-old to be 80 years old and above.

Each patient’s record was accessed, and data was manually extracted from clinical documentation, review of results and prescription. Prior to data extraction, study members underwent one training session. Throughout the data collection period, disputes were discussed regularly. Data was collected in 4 parts: demographic characteristics, clinical indices, healthcare utilisation and quality of end-of-life care. Demographic information included: age, sex, race, marital status, admission ward class, primary residence and social setup including home services and caregivers. Clinical indices included Charlson Comorbidity Index (CCI) [21] and frailty by FRAIL-NH using incontinence. FRAIL-NH score of 0–1 is robust, 2–6 pre-frail and 7 or above as frail [22]. The FRAIL-NH scale has been validated in Asians populations including Korea [23]. Locally, FRAIL has been compared to other frailty measurements such as the Clinical Frailty Scale and Tilburg Frailty Indicator, and has been found to be better at predicting in-hospital mortality and length of hospitalisation [24]. However, this study looked at the more robust inpatient older adults whereas our population is towards end-of-life, and hence our study team decided on the variation for frailer older adults. Polypharmacy was defined as 5 or more medications. Data on functional status was collected.

For outcome measures, healthcare utilisation was assessed by total number of days spent in hospital, number of hospital admissions, length of stay of the terminal admission, ED visits and specialist outpatient clinic visits. There is no standardised quality of care measurement for terminal care, though there is consensus to examine two main groups of outcomes: life processes and end-of-life outcomes [25]. Our markers is a composite of previously used tools [26–28]. Quality of end-of-life care included number of investigations, symptom control, status of advanced care planning (ACP) and quality of communication with patients and their family. For radiological investigations, we counted the total number of common scans done for the last admission. These include: X-rays (chest, abdominal, spine, hip, kidney and urinary bladder), computerized tomography (CT) (brain, pulmonary angiogram, abdomen-pelvis) and magnetic resonance imaging (MRI) scans (brain, spine).

Descriptive analyses were carried out. Pearson’s chi-square test was used for categorical variables and independent sample *t*-test was used for continuous variables. As the outcomes are count data, the negative binomial distribution was used to model all outcomes due to overdispersion. The other variables (sex, CCI, place of residence, home service, frailty and dementia) were adjusted as confounders associated with of healthcare utilisation beyond that of age. All tests of significance used the 95% level (*p* < 0.05). All analyses were performed using Stata 14.0 (StataCorp. 2015. *Stata Statistical Software: Release 14*. College Station, TX: StataCorp LP). Results were reported to 1 decimal place.

## Results

Among the decedents, 189 records were examined. Healthcare demographics and comparison indices are shown in Table 1.

**Table 1:** Comparison of indices between young-old and old-old decedents

	Total (*n* = 189)	Young-Old (< 80 years old) (*n* = 57)	Old-Old (≥80 years) (*n* = 132)	*P* value
**Demographics**				
Age* (mean, SD)	84.3 (8.6)	73.8 (4.0)	88.9 (5.5)	**< 0.001**
Sex* (count, %)				**0.004**
Female	107 (56.6)	24 (42.1)	83 (62.9)	
Male	82 (43.4)	33 (57.9)	49 (37.1)	
**Residence (count, %)**				0.07
Home	142 (75.1)	38 (66.7)	104 (78.8)	
Nursing home	47 (24.9)	19 (33.3)	28 (21.2)	
Primary Caregiver* (count, %)				**0.001**
Self	17 (9.0)	10 (17.5)	7 (5.3)	
Spouse	7 (3.7)	3 (5.3)	4 (3.0)	
Child	22 (11.6)	3 (5.3)	19 (14.4)	
FDW	89 (47.1)	19 (33.3)	70 (53.0)	
Others	54 (28.6)	22 (38.6)	32 (24.3)	
**Home services** (count, %)	37 (19.6)	13 (22.8)	24 (18.2)	0.50
**Ambulation status** (count, %)				0.80
Walking aid	37 (19.9)	13 (21.0)	24 (18.5)	
Wheelchair bound	30 (16.1)	4 (6.5)	26 (20.0)	
Bedbound	119 (64.0)	39 (69.6)	80 (61.5)	
**Clinical Indices**				
**FRAIL-NH** (mean, SD)	7.3 (3.4)	7.0 (3.9)	7.5 (3.2)	0.30
CCI* (mean, SD)	7.5 (2.5)	6.9 (2.8)	7.7 (2.4)	**0.007**
**Dementia** (count, %)	79 (41.8)	21 (36.8)	58 (43.9)	0.30
**Polypharmacy** (count, %)	124 (65.6)	41 (71.9)	83 (62.9)	**0.01**
**Commonly used medications** (count, %)				
Statin	32 (17.0)	8 (14.0)	24 (18.3)	0.5
Aspirin	36 (19.2)	10 (17.5)	26 (20.0)	0.7
PPI	63 (33.3)	21 (36.8)	42 (31.8)	0.5

*FDW* foreign domestic worker, *FRAIL-NH* frailty score, *CCI* Charlson Comorbidity Index, *ACP* advance care planning, *ED* emergency department, *PPI* proton pump inhibitor;

* denotes *p* < 0.05

Of the 189 decedents, 132 (70%) belonged to the old-old group. Old-old decedents tend to be females (63% vs. young-old 42%, *p* = 0.004), have foreign domestic workers (FDW) as primary caregiver (53% vs. young-old 33%, *p* = 0.001), higher CCI scores (7.7 vs young-old 6.6, *p* = 0.007), less likely to have polypharmacy (63% vs. 73%, *p* = 0.01), have shorter length of stay in the last year of life (22 vs. 30.5 days, *p* = 0.04) and fewer clinic visits (2.3 vs 3.7 days, *p* = 0.03) (Table 1).

Table 2 details end-of-life care in both groups. There are more radiological studies in young-old (5.2 ± 8.3 vs 3.1 ± 2.8, *p* = 0.012). Both groups had 6 to 10 radiological studies done in their last admission. Antibiotics use was high in both groups, with 93% in young-old and 94.7% in old-old. Symptom control was similar in both groups.

**Table 2:** Outcome measures

	Total (*n* = 189)	Young-Old (< 80 years old) (*n* = 57)	Old-Old (≥80 years) (*n* = 132)	*P* value
**Healthcare utilisation**				
**Total number of hospital admissions** (mean, SD)	2.6 (2.0)	3.0 (2.2)	2.5 (1.9)	0.12
Total number of days spent in hospital during last year of life* (mean, SD)	24.8 (28.1)	30.5 (37.7)	22.3 (22.4)	**0.04**
**ED admission** (mean, SD)	2.7 (2.0)	3.0 (2.2)	2.6 (1.9)	0.20
Clinic visits* (mean, SD)	2.6 (4.1)	3.4 (4.9)	2.3 (3.2)	**0.03**
**Length of stay in terminal admission** (mean, SD)	8.5 (8.4)	9.3 (9.0)	8.1 (8.1)	0.20
**End-of-life care**				
Radiological studies* Number (mean, SD)	3.7 (5.2)	5.2 (8.3)	3.1(2.8)	**0.012**
1 (count, %)	58 (30.7)	16 (2.8)	42 (31.8)	
2–5	102 (54.0)	27 (47.4)	75 (56.8)	
6–10	18 (9.5)	7 (12.3)	11 (8.3)	
> 10	15 (7.9)	12 (21.1)	3 (2.3)	
**Antibiotics** Use (count, %)	178 (94.2)	53 (93.0)	125 (94.7)	0.93
Number of antibiotics (mean, SD)	1.8 (1.0)	1.8 (1.1)	1.8 (1.0)	
**Symptom control** (count, %)				
Opiates	128 (67.7)	43 (75.4)	85 (64.4)	0.14
Sedatives	15 (7.9)	6 (10.5)	9 (6.8)	0.38
Anti-cholinergic	58 (30.7)	19 (33.3)	39 (29.6)	0.61
Anti-emetics	8 (4.3)	2 (3.5)	6 (4.6)	0.74
**ACP** (count, %)				
Referred	31 (16.4)	10 (17.5)	21 (15.9)	0.70
Completed (out of referred ACP)	10 (32.3)	2 (20.0)	8 (38.1)	0.7

*ED* emergency department, *ACP* advance care planning;

* denotes *p* < 0.05

The results of the negative binomial model on the incremental utilisation associated with age group, adjusted for socio-demographic characteristics, comorbidities and clinical measures are shown in Table 3. When adjusted for sex, comorbidity and frailty, old-old decedents spent 7.3 (3.5) less days in hospital in their final year of life, significantly lower than young-old. With further adjustment for dementia and primary residence, old-old also had 0.5 (0.3) less hospital admissions.

**Table 3:** Incremental effect of healthcare utilisation during the last year of life by age group, adjusting for socio-demographic characteristics, comorbidities and clinical measures

Model	Variable	Healthcare utilisation measures (incremental effect, SE)				
		Total number of hospital bed days	Length of stay	ED admissions	Total number of admissions	Total number of clinic visits
Model 1	Age 80 years old and above	−7.257* (3.51)	−0.630 (1.26)	−0.388 (0.26)	− 0.505 (0.27)	− 1.952 (1.79)
Model 2	Age 80 years old and above	− 7.100* (3.57)	− 0.838 (1.02)	− 0.421 (0.25)	− 0.523* (0.26)	− 2.058 (1.45)
Model 3	Age 80 years old and above	− 7.099* (3.49)	−1.032 (0.80)	−0.390 (0.28)	− 0.505 (0.28)	− 2.059 (1.47)

*ED* emergency department, *CCI* Charlson comorbidity index;

*p < 0.05;

Model 1 adjusted for age, sex, CCI, FRAIL-NH;

Model 2 adjusted for age, sex, CCI, FRAIL-NH, dementia, primary residence;

Model 3 adjusted for age, sex, CCI, FRAIL-NH, dementia, primary residence, home services

Model 3 (Table 4) revealed 2.7 (0.8) days shorter length of stay in the final admission in old-old who were recipients of home care services. This significant reduction persisted in all recipients of home care regardless of age (young-old − 3.1 (1.5) days vs old-old − 3.3 (1.4) days).

**Table 4:** Model 3

Variables	Total number of hospital bed days	Length of stay	ED admissions	Total number of hospital admissions	Total number of clinic visits
Age (ref young-old)	−7.099* (3.49)	−1.032(0.8)	−0.390 (0.28)	− 0.505 (0.28)	−2.059 (1.47)
Sex (ref female)	1.669 (3.03)	0.580 (0.73)	0.597* (0.26)	0.489 (0.26)	0.901 (1.24)
CCI (ref CCI < 5)	11.537** (3.62)	2.318** (0.89)	0.652* (0.33)	0.833* (0.33)	1.316 (1.45)
Place of residence (ref home)	1.622 (3.77)	−3.095*** (0.81)	−0.186 (0.30)	− 0.143 (0.31)	−4.172** (1.58)
Home service (ref none)	−0.908 (3.85)	−2.743** (0.83)	0.413 (0.35)	0.38 (0.36)	0.012 (1.64)
FRAIL-NH (ref robust 0–1)					
Prefrail (2–5)	4.268 (5.42)	−2.258 (1.71)	0.894 (0.49)	0.576 (0.51)	−1.487 (3.22)
Frail (> 5)	7.848 (4.84)	−1.586 (1.72)	0.861 (0.43)*	0.525 (0.45)	−2.454 (3.20)
Dementia (ref none)	−0.895 (3.00)	−0.922 (0.75)	0.132 (0.26)	0.003 (0.26)	1.087 (1.40)
Observations	189	189	189	189	189

*ED* emergency department, *CCI* Charlson comorbidity index;

* *p* < 0.05, ** *p* < 0.01, *** *p* < 0.001

**Fig. 1:**
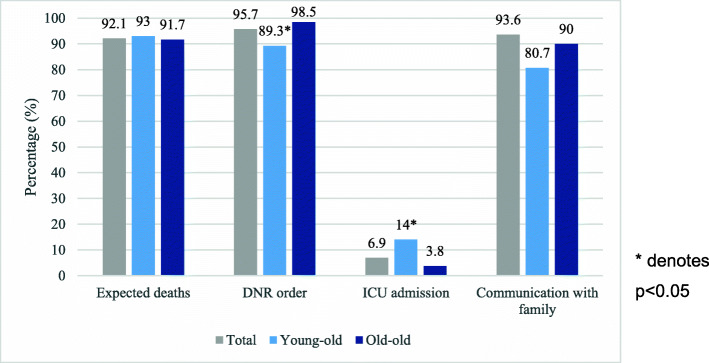
Bar chart of communication during end-of-life. * denotes *p* < 0.05. *DNR* do not resuscitate, *ICU* intensive care unit

Among the decedents, 47 (24.4%) had a decrease in baseline function from previous admissions and only 54 (28.6%) were communicative. 174 (92.1%) deaths were expected, 179 (95.7%) had a “do not resuscitate” (DNR) order and 13 (6.9%) entered ICU from the general ward. Whilst in a large majority of cases communication of poor prognosis to the family was documented, in 12 (6.4%) patients this was not carried out. Significantly less young-old had a DNR status (*p* = 0.0043) and correspondingly more young-old entered ICU (*p* = 0.01) compared to old-old (Fig. 1). Use of palliative care services were low, only in 6 cases.

## Discussion

Our study is one of the few studies looking at healthcare utilisation in the last year of life in mainly non-cancer patients with unpredictable trajectory and recovery. With longer life expectancy, we are beginning to see the heterogeneity between the young-old and old-old [29, 30]. In our study, as expected, there were more females in the old-old group as females tend to have a longer life expectancy [2]. Both groups were similarly frail and experienced similar incidence of dementia. CCI scores were higher in old-old which is expected as age is a variable in the scoring. There was higher healthcare utilisation in the young-old despite the lack of difference in functional status and frailty between the two groups, although prevalence of dementia was non-significantly higher in the old-old group. This has been observed in previous studies [31, 32]. Specifically, our old-old had less days spent in hospital during their last year of life, fewer number of hospital admissions, clinic visits and lower prevalence of polypharmacy.

There have been conflicting healthcare utilisation patterns seen in our older adults. This is a product of healthcare systems, societal values and caregiving setup as much as it is due to disease and their associated morbidities. Though the healthcare setup differ between countries, there are similarities: depleted savings from years of retirement and ill health, cultural stoicism and reluctance for medical care [33]. These factors all contribute to our old-old not seeking medical attention, which can lead to delays or suboptimal treatment and subsequent caregiver breakdown. Our findings correspond closely to Arivalagan and Gee’s report [13] looking at perspectives of healthcare in our older adult and their social ecosystem. For Asian patients, families play a bigger, if not dominant, role in healthcare utilisation, with older adults often deferring decision-making capacity to family especially if they rely on them for financial support [13]. In terms of psychological factors, feelings of being a burden to family, perceived unnecessary expense on health coupled with Singapore’s co-payment healthcare system, and reluctance to engage with Western medicine are common reasons in our old-old. On the other hand, our young-old may be more receptive to the hospital care as they come from an era when Western medicine was becoming commonplace in the society. With the constant shift in intergenerational differences, care would have to continually adapt to maintain relevance in our older adults’ cultural context.

Physician preference also plays a significant role. There is a complex relationship between physician and patient preferences of care. In a previous study, there was good correlation in physician-patient preferences despite lack of explicit conversation, suggesting inter-influence [34]. In our young-old, physicians may be more aggressive as they may be more hopeful for recovery, especially for those who at premorbid are self-caring. Our young-old may reflect this by wanting more aggressive care as well. Conversely, physicians, patients and family may perceive more risk of adverse effects and lower benefit in the old-old, resulting in less interventions and healthcare contact. To our knowledge, there has been no formal studies looking at physician preference by patient ages. However, literature review shows the clinical reality, reflecting persistent use of chronological age in literature [35] to define treatment groups. This is in spite of several studies calling for an individualised approach to tailor to the older adults’ heterogeneity, with varying degrees of frailty and corresponding differing healthcare utilisation patterns [29, 30]. Our data illustrates that physicians do still tend to place emphasis on chronology more than biology, and a change is sorely due to tailor effective approaches for our individually unique older adult.

Beyond understanding the heterogeneity of our older adults, to optimise healthcare utilisation at the end-of-life, we have to target delivery. This rests on two premises: timing of recognition and communication. Due to the non-predictable trajectory in non-cancer patients, recognition that patient is approaching end-of-life and its related communication becomes a challenge. Neither frailty, functional status nor comorbidity indices were useful in predicting recurrent admissions to hospital in the terminal phase, unlike in studies done in Hong Kong [36] and Europe [37]. The significant difference in CCI scoring seen in our study is in part due to age being a scoring factor. It may then be hard to time recognition earlier, and much of this rests on experience of the clinician.

Our review of documentation found that a vast majority of healthcare teams did recognise the expected deaths and did communicate with family, suggesting that this may not be the key problem. Recognition however did not translate to action. This is where the details of communication come in. Whilst our study shows that communication did take place, exploration of extent on investigations, medications and interventions is lacking. Only half had discontinuation of unwarranted investigations and more than 90% died on antibiotics, which is higher than use in hospitalised oncology patients in their last week of life [38]. The timing of advance care planning is also crucial as only 28.6% of patients were communicative in their terminal admission and the majority would have been unable to express their wishes. Our data does show that we are missing on earlier opportunities to broach ACP: we have low ACP uptake and most often they are not completed as patient had demised. This finding can be explained by the low level of physician awareness of when to start the conversation about ACP. In its current state, ACP is often done reactively, triggered after multiple readmissions, rather than proactively by identifying that the patient is at risk of further decline. Many factors contribute to this, including reluctance to acknowledge impending mortality with lack of objective accurate prognostic tools, diffusion of responsibility, limited clinician time and differing opinions of ACP [39]. This is unfortunate as ACP is a powerful health behaviour tool that provides a structured approach to starting the conversation at an early stage, and it can be easily tailored to the current health condition whilst opening discussion for potential eventualities [40]. This way, the patient can play an active role instead of the current situation of playing catch-up and relying on family members as proxy. This is in contrast to communication on the wards at present, where though death is predicted and conversation is carried out, due to the nature of an acute admission, planning is short sighted and does not explore options for the next readmission or care at home. ACP has been shown to reduce days spent in hospital in the last year of life [41]. There is also good evidence to show that ACP is helpful in improving patient and family satisfaction and alleviating anxiety during admission [42]. But first, ACP has to be used in a timely fashion to have any effect.

We have made much progress in our palliative care domain over recent years. Such services include home-based care such as hospice, medical and nursing support as well as facilitators for ACP. Our data shows that with home service our older adults spend less days in hospital during their terminal admission. It is likely that having home services allows the older adult to stay at home longer. Studies have shown conflicting results: some identify home service to be a cost-effective way to reduce readmission rates [43], whereas others led to more ED visits [44] needing better matching of recipient needs and home service provision [45]. From our study, home service uptake is poor across both groups. There are many reasons for this, such as cost, reluctance for strangers into the house and lack of access. We need to develop this service further.

Notably, only 6 decedents received specialist palliative care. This low number of referrals is echoed internationally [46, 47]. Given the increasing number of patients suffering and dying from non-cancer diseases [48, 49], this phenomenon is worrying. Many barriers still exist [47], such as the unpredictable non-cancer trajectory, the resultant difficulties in forming a referral criterion and the lack of non-cancer disease expertise.

To facilitate these changes, we need to enhance education and awareness of palliative care amongst healthcare professionals and the community [13]. By increasing the presence of palliative care in the community and normalising conversations, the stigma of talking about death can be slowly but surely chipped away, not just our patients but also among our healthcare colleagues [50].

### Strengths and limitations

Our study’s strength lies in its examination of mainly non-cancer patients with multiple comorbidities, minimally communicative and two-thirds being bedridden with unpredictable trajectory. As majority of the older non-cancer patients receiving end-of-life care were under medicine, we did not examine the surgical population in our study. Furthermore, as our study seeks to look at healthcare utilisation, our inclusion criteria selected for those sick enough to need hospital level care, and patients who were well managed by outpatient or community services were omitted.

Our study team decided on a retrospective design as non-cancer death is difficult to prognosticate which makes a prospective study logistically challenging without prior data. There is also relatively less data available for this population. Our small study hence aims to be exploratory. Confounding variables include variable amount of palliative medicine training of hospitalists in charge of patient’s care, expertise of the ward nurse in managing terminal care and quality of documentation. Our variables and outcomes are correlational. As confounding bias cannot be completely eliminated; we cannot draw conclusions on causal relationship.

We applied FRAIL-NH to our study as we were focussing on older adults in their last year of life. A sample study of our first ten cases found that most patients in our cohort fell within the demographic more in-fitting of long-term care. However, whereas FRAIL has been validated in our local population, FRAIL-NH has only been validated in similar Asian populations such as Korea. The main differences between the FRAIL and FRAIL-NH scale are in indices of resistance, ambulation, being able to use incontinence instead of illnesses, addition of nutrition and help with dressing. Assessing use of stairs and aerobic activities in our population would not be discriminatory as nearly all of them were not able to do so. Similarly, most had more than five illnesses. Hence to obtain granularity on the degree of frailty, we decided to use the FRAIL-NH scale instead of the FRAIL scale. This difference is likely due to our local care structure of significant family involvement and having domestic helpers as informal caregivers. This caregiving system allows many fully dependent patients to be cared for at home rather than in institutions. Our findings also reflect this: more than 60% of patients were bedbound, but most of these patients were still able to be cared for at home.

As the goal of our study was for characterisation, we also did not include a control group involving patients who did not die during the same time period. Hence, modelling of variables for predicting high recurrence during the last year of life was not possible. We present trends noted, though they are not causal. As non-cancer deaths are relatively uncommon compared to cancer deaths, we started with a retrospective design to obtain baseline data. We hope this can inspire prospective studies looking into non-cancer related end-of-life care.

## Conclusion

Our young-old had significantly higher healthcare utilisation than that of our old-old despite similar functional and frailty status. There was high prevalence of polypharmacy, investigations and antibiotics prescription in this group with low ACP uptake. With better communication, taking into account frailty and functional status, more cost-effective care at home can be provided for this group of patients. ACP is a powerful tool in adapting the end-of-life conversation and could assist in better care planning. However, enhanced education of healthcare professionals is needed in order to increase uptake of ACP. Shifting the focus away from chronological age and into biological frailty would help target our resources and provide cost-effective quality care. We are in an age of great awareness with many effective tools at our disposal; with knowledge, we can wield them in a way to ensure our patients a good death.

## Data Availability

The datasets used and/or analysed during the current study are available from the corresponding author on reasonable request.

## Abbreviations

ACP: Advanced care planning
AIM: Advanced Internal Medicine
CCI: Charlson Comorbidity Index
CT: Computer tomography
DNR: Do not resuscitate
ED: Emergency department
ICU: Intensive care unit
MRI: Magnetic resonance imaging
NUH: National University Hospital

## References

[1] Bank W. Live long and prosper : aging in East Asia and Pacific. World Bank East Asia and Pacific Regional Report: Washington, DC. *©*; 2016.

[2] Department of Statistics. Death and Life Expectancy. 2020. Available from: https://www.singstat.gov.sg/find-data/search-bytheme/population/death-and-life-expectancy/latest-data. Accessed Nov 2020.

[3] Boon TT. A super-aged Singapore: Policy implications for a Smart Nation. 2015. Available from: https://www.rsis.edu.sg/rsispublication/rsis/co15193-a-super-aged-singapore-policy-implications-for-a-smart-nation/#.X7OliGgzbHo. Accessed Nov 2020.

[4] East Asia Forum, 2015. Can a Rapidly Aging Singapore Stave Off Economic Disaster? Available from: http://www.economywatch.com/features/Can-a-Rapidly-Aging-Singapore-Stave-Off-Economic-Disaster1005.html. Accessed Nov 2020.

[5] Ministry of Health. Government Health Expenditure. 2020. Available from: https://data.gov.sg/dataset/government-healthexpenditure. Accessed Nov 2020.

[6] Chen C, Chia NC, Wang L, Tysinger B, Zissimopolous J, Chong MZ, Wang Z, Koh GC, Yuan JM, Tan KB, Chia KS, Cook AR, Malhotra R, Chan A, Ma S, Ng TP, Koh WP, Goldman DPYJ, L.J (2019). The long-term impact of functional disability on hospitalization spending in Singapore. The Journal of the Economics of Ageing.

[7] Calver J, Bulsara MBD. In-patient hospital use in the last years of life: a Western Australian population-based study. Aust N Z J Public Heal. 2006;(30):143–6. 10.1111/j.1467-842x.2006.tb00107.x 16681335

[8] Moorin REHC (2008). The cost of in-patient care in western Australia in the last years of life: a population-based linkage study. Health Policy (New York).

[9] Gielen B, Remacle AMR (2010). Patterns of health care use and expenditure during the last 6 months of life in Belgium: differences between age categories in cancer and non-cancer patients. Health Policy (New York).

[10] Evans CJ, Ho Y, Daveson BA, Hall S, Higginson IJGWO, behalf of the G project (2014). Place and cause of death in centenarians: a population-based observational study in England, 2001–2010. PLoS Med.

[11] Teno JM, Gozalo P, Trivedi AN, Bunker J, Lima J, Ogarek JMV (2018). Site of death, place of care, and health care transitions among US medicare beneficiaries, 2000–2015. Jama.

[12] Salomon JA, Wang H, Freeman MK, Vos T, Flaxman AD, Lopez ADMC (2012). Healthy life expectancy for 187 countries, 1990-2010: a systematic analysis for the global burden disease study 2010. Lancet..

[13] Arivalagan YGC (2019). Leaving well: end-of life policies in Singapore.

[14] Lunney JR, Albert SM, Boudreau R, Ives D, Satterfield S, Newman AB, HTHA and BCS (2018). Mobility trajectories at the end of life: comparing clinical condition and latent class approaches. J Am Geriatr Soc.

[15] Mounsey L, Ferres MEP (2018). Palliative care for the patient without cancer. Aust J Gen Pr.

[16] Lastrucci V, D’Arienzo S, Collini F, Lorini C, Zuppiroli A, Forni S, Bonaccorsi G, Gemmi FVA (2018). Diagnosis-related differences in the quality of end-of-life care: a comparison between cancer and non-cancer patients. PLoS One.

[17] Koffman J, Yorganci E, Yi D, Gao W, Murtagh F, Pickles A, Barclay S, Johnson H, Wilson R, Sampson L, Droney J, Farquhar M, Prevost TEC (2019). Managing uncertain recovery for patients nearing the end of life in hospital: a mixed-methods feasibility cluster randomised controlled trial of the AMBER care bundle. Trials..

[18] Carey EC, Dose AM, Humeniuk KM, Kuan YC, Hicks AD, Ottenberg AL (2018). The experience of hospital death: assessing the quality of care at an Academic Medical Center. Am J Hosp Palliat Med.

[19] Statistics Singapore. Old-age Support Ratio. 2020. Available from: https://www.singstat.gov.sg/modules/infographics/old-agesupport-ratio. Accessed Nov 2020.

[20] Nations U (2015). World population ageing..

[21] Charlson ME, Pompei P, Ales KL (1987). A new method of classifying prognostic comorbidity in longitudinal studies: development and validation. J Chronic Dis.

[22] Morley JE, Malmstrom TKMD (2012). A simple frailty questionnaire (FRAIL) predicts outcomes in middle aged African Americans. J Nutr Health Aging.

[23] Ga H, Won CWJE (2018). Use of the frailty index and FRAILNH scale for the assessment of the frailty status of elderly individuals admitted in a long-term care hospital in Korea. Ann Geriatr Med Res.

[24] Chong E, Ho E, Baldevarona-Llego J, Chan M, Wu L, Tay L (2018). Frailty in hospitalized older adults: comparing different frailty measures in predicting short- and long-term patient outcomes. J Am Med Dir Assoc.

[25] Waller A, Dodd N, Tattersall MHN, Nair BS-FR (2017). Improving hospital-based end of life care processes and outcomes: a systematic review of research output, quality and effectiveness. BMC Palliat Care.

[26] Miyashita M, Nakamura A, Morita TBS (2008). Identification of quality indicators of end-of-life cancer care from medical chart review using a modified Delphi method in Japan. Am J Hosp Palliat Care.

[27] Lorenz KA, Dy SM, Naeim A (2009). Quality measures for supportive cancer care: the cancer quality ASSIST project. J Pain Symptom Manag.

[28] Service NH (2011). End of Life Care Quality Assessment (ELCQuA).

[29] Lowsky DJ, Olshansky SJ, Bhattacharya JGD (2014). Heterogeneity in healthy aging. J Gerontol A Biol Sci Med Sci.

[30] Lee SB, Oh JH, Park JH, Choi SPWJ (2018). Differences in youngest-old, middle-old, and oldest-old patients who visit the emergency department. Clin Exp Emerg Med.

[31] Zhu B, Li F, Wang C, Wang L, He Z, Zhang X, Song P, Ding LJC (2018). Tracking hospital costs in the last year of life - the Shanghai experience. Biosci Trends.

[32] Wammes JJG, van der Wees PJ, Tanke MAC, Westert GPJP (2018). Systematic review of high-cost patients’ characteristics and healthcare utilisation. BMJ Open.

[33] Moore A, Grime J, Campbell PRJ (2013). Troubling stoicism: sociocultural influences and applications to health and illness behaviour. Heal..

[34] Gramelspacher GP, Zhou XH, Hanna MPTW (1997). Preferences of physicians and their patients for end-of-life care. J Gen Intern Med.

[35] Sabharwal S, Wilson H, Reilly PGC (2015). Heterogeneity of the definition of elderly age in current orthopaedic research. Springerplus..

[36] J. WJ and L (2014). Multi-morbidity, dependency, and frailty singly or in combination have different impact on health outcomes. Age..

[37] S. IS and C (2015). The patterns of health care utilization by elderly Europeans: frailty and its implications for health systems. Health Serv Res.

[38] Thompson AJ, Silveira MJ, Vitale CAMP (2012). Antimicrobial use at the end of life among hospitalized patients with advanced cancer. Am J Hosp Palliat Care.

[39] Scott IA, Mitchell GK, Reymond EJDM (2013). Difficult but necessary conversations--the case for advance care planning. Med J Aust.

[40] Fried TR, Redding CA, Robbins ML, Paiva AL, O’Leary JRIL (2016). Development of personalized health messages to promote engagement in advance care planning. J Am Geriatr Soc.

[41] Abel J, Pring A, Rich A, Malik TVJ (2013). The impact of advance care planning of place of death, a hospice retrospective cohort study. BMJ Support Palliat Care 2013.

[42] Detering KM, Hancock AD, Reade MCSW (2010). The impact of advance care planning on end of life care in elderly patients: randomised controlled trial. BMJ..

[43] Roeper B, Mocko J, O’Connor LM, Zhou J, Castillo DBE (2018). Mobile integrated healthcare intervention and impact analysis with a medicare advantage population. Popul Heal Manag.

[44] Jones A, Schumacher C, Bronskill SE (2018). The association between home care visits and same-day emergency department use: a case-crossover study. CMAJ..

[45] Gruneir A, Fung K, Fischer HD (2018). Care setting and 30-day hospital readmissions among older adults: a population-based cohort study. CMAJ..

[46] Stiel S, Heckel M, Seifert A, Frauendorf T, Hanke RMOC. Comparison of terminally ill cancer- vs. non-cancer patients in specialized palliative home care in Germany - a single service analysis. BMC Palliat Care. 2015;14(34). 10.1186/s12904-015-0033-z PMC4514986 26209094

[47] O’Leary NTE (2008). Survey of specialist palliative care services for noncancer patients in Ireland and perceived barriers. Palliat Med.

[48] Solano JP, Gomes BHI (2006). A comparison of symptom prevalence in far advanced cancer, AIDS, heart disease, chronic obstructive pulmonary disease and renal disease. J Pain Symptom Manag.

[49] Murtagh FE, Preston MHI (2004). Patterns of dying: palliative care for non-malignant disease. Clin Med.

[50] DE Prince-Paul M (2017). Upstreaming and normalizing advance care planning conversations—a public health approach. Behav Sci (Basel).

